# The extracellular matrix integrates mitochondrial homeostasis

**DOI:** 10.1016/j.cell.2024.05.057

**Published:** 2024-06-27

**Authors:** Hanlin Zhang, C. Kimberly Tsui, Gilberto Garcia, Larry K. Joe, Haolun Wu, Wudi Fan, Sentibel Pandovski, Peter Yoon, Brant Michael Webster, Jenni Durieux, Phillip Andrew Frankino, Ryo Higuchi-Sanabria, Andrew Dillin

**Affiliations:** 1 Department of Molecular & Cellular Biology, Howard Hughes Medical Institute, University of California, Berkeley, Berkeley, CA 94720, USA; 2 Present address: Leonard Davis School of Gerontology, University of Southern California, Los Angeles, CA 90089, USA; 3 Lead Contact

**Keywords:** Extracellular matrix, hyaluronan, TMEM2, mitochondria, TGF-β, immunity, longevity

## Abstract

Cellular homeostasis is intricately influenced by stimuli from the microenvironment, including signaling molecules, metabolites, and pathogens. Functioning as a signaling hub within the cell, mitochondria integrate information from various cellular compartments to regulate cellular signaling and metabolism. However, it is unknown how changes in the cellular microenvironment, particularly the extracellular matrix (ECM), can impact mitochondrial homeostasis. We find that ECM remodeling alters mitochondrial homeostasis in an evolutionarily conserved manner. Mechanistically, ECM remodeling triggers a TGF-β response to induce mitochondrial fission and the unfolded protein response of the mitochondria (UPR^MT^). Interestingly, ECM remodeling promotes immunity of animals towards pathogens through the enhanced mitochondrial stress responses. We postulate that this ECM-mitochondria crosstalk represents an ancient immune pathway, which detects infection- or mechanical stress-induced ECM damage, thereby initiating adaptive mitochondria-based immune and metabolic responses.

## Introduction

Cells are continually having to sense and communicate aspects of their extracellular environment to the inside of the cell. Many of these sensing mechanisms require ligand-receptor interactions at the plasma membrane to elucidate the proper cellular response. However, each cell is coated in a thick structure, the ECM, composed of proteins and large carbohydrates. In fact, the ECM can be up to 600 nm thick, thus posing a huge challenge on any ligand-receptor interaction, especially those involving pathogen detections systems. Therefore, could there be communication events dictated from the ECM to inform the inside of the cell about possible challenges on the outside of the cell? If so, what could be the source of this communication, what is communicated, and finally, what changes on the inside of the cell are created to match the external challenges? In this study, we applied genetic approaches to remodel the ECM in mammalian cells and *C. elegans*, unraveling the molecular mechanisms of an ECM-mitochondria communication and the physiological significance of this newly identified inter-organellar communication in live animals.

The ECM is the major component of the extracellular microenvironment where intercellular and host-microbiome communications occur. In general, the ECM comprises numerous components that are constantly renewed, including large protein fibers, glycosaminoglycans (GAGs), and glycoproteins. The ECM is a frequent target of viruses, bacteria, and fungi to achieve successful adhesion, invasion, and pathogenesis. For example, the human opportunistic pathogenic bacteria *E. faecalis* expresses multiple adhesins, such as collagen-binding proteins, and invasins, such as hyaluronidases and gelatinases, that contribute to their virulence.^1^

Hyaluronan (hyaluronic acid, HA) is a non-sulfated GAG that is a major component of the mammalian ECM. It is a linear polymer composed of repeating disaccharide units of glucuronic acid and *N*-acetyl-glucosamine. Under steady conditions, HA exists in a high-molecular-weight (HMW) format reaching 2,000 kDa, approximately 6,000 disaccharide units in human tissues^2^. HA fibers maintain structural integrity of the ECM and regulate cellular behaviors, such as migration and repair.^3^ The size of HA fibers is a balance between hyaluronan synthases and hyaluronidases. The transmembrane protein TMEM2 is a major cell-surface hyaluronidase that degrades HMW HA into low-molecular weight (LMW) ~5 kDa fragments.^4–6^ Notably, TMEM2’s cleavage activity is specific to non-sulfated HA and does not cleave other sulfated GAGs.^4^ Besides degradation caused by pathogen-derived external hyaluronidases, accelerated degradation of HA is found in other pathologic conditions including rheumatoid arthritis, diabetes, cancer, and inflammation,^3,7^ possibly caused by altered cellular HA metabolism. As such, HA degradation is a key marker and contributing catalyst in the progression of diseases, but its precise implications for cellular physiology are unknown.

In addition to providing mechanical support for the cell, the ECM also functions as a reservoir of signaling molecules. Degradation products from large ECM molecules, such as endostatin, derived from the cleavage of collagen XVIII, regulates immune responses, fibrosis, and cell proliferation.^8,9^ Although HA has a relatively simple structure, a variety of HA-binding proteins (HABPs) have been identified, such as CD44 and TLR4, whose activities may be affected upon binding to HA.^10^ Specifically, LMW HA fragments are pro-inflammatory and trigger cell migration and proliferation, whereas HMW HA promotes anti-inflammatory and antiproliferative effects.^3^ Besides molecules directly derived from cleavage of large ECM polymers, small secreted signaling molecules, such as growth factors and cytokines, are found – and sometimes even enriched – in the ECM.^11^ As a result, disturbance of the ECM can modulate the signaling activities of these molecules. For example, TGF-β ligands are secreted and stored in the ECM in their latent forms. Upon mechanical or biochemical stimuli that disrupts the ECM, TGF-β ligands are released from the ECM and signal via the downstream SMAD pathways to initiate a series of cellular responses such as proliferation, differentiation, apoptosis, and ECM deposition.^12^

While the ECM can coordinate signaling events outside of the cell, mitochondria are crucial signaling hubs inside of the cell. Mitochondria direct cellular metabolism, stress responses, innate immunity, and cell death.^13^ Orchestrating mitochondrial homeostasis involves intricate mechanisms, including balanced expression of mitochondrial genes encoded by the nuclear and mitochondrial genomes, mitochondrial fission and fusion, mitochondrial stress responses including the UPR^MT^ and the oxidative stress response that restore mitochondrial homeostasis via promoting transcription of cellular homeostatic proteins, and mitophagy that removes damaged mitochondria. Activation of mitochondrial stress responses enhance organismal resistance to environmental stress and aging.^14^ The concept of leveraging mild mitochondrial stress, known as mitohormesis, to trigger protective responses for improved resilience has garnered extensive attention.^15^ Alongside mitochondrial toxins, physiological inducers of mitochondrial stress include redox status changes, mitochondrial gene mutations, and altered interactions with other organelles. While it is plausible that mitochondrial form and function could align with cellular behavior adjustments prompted by extracellular stimuli, the role of the ECM in these processes has not been explored.

## Results

### ECM remodeling results in altered mitochondrial function in human cells

**Figure 1.**
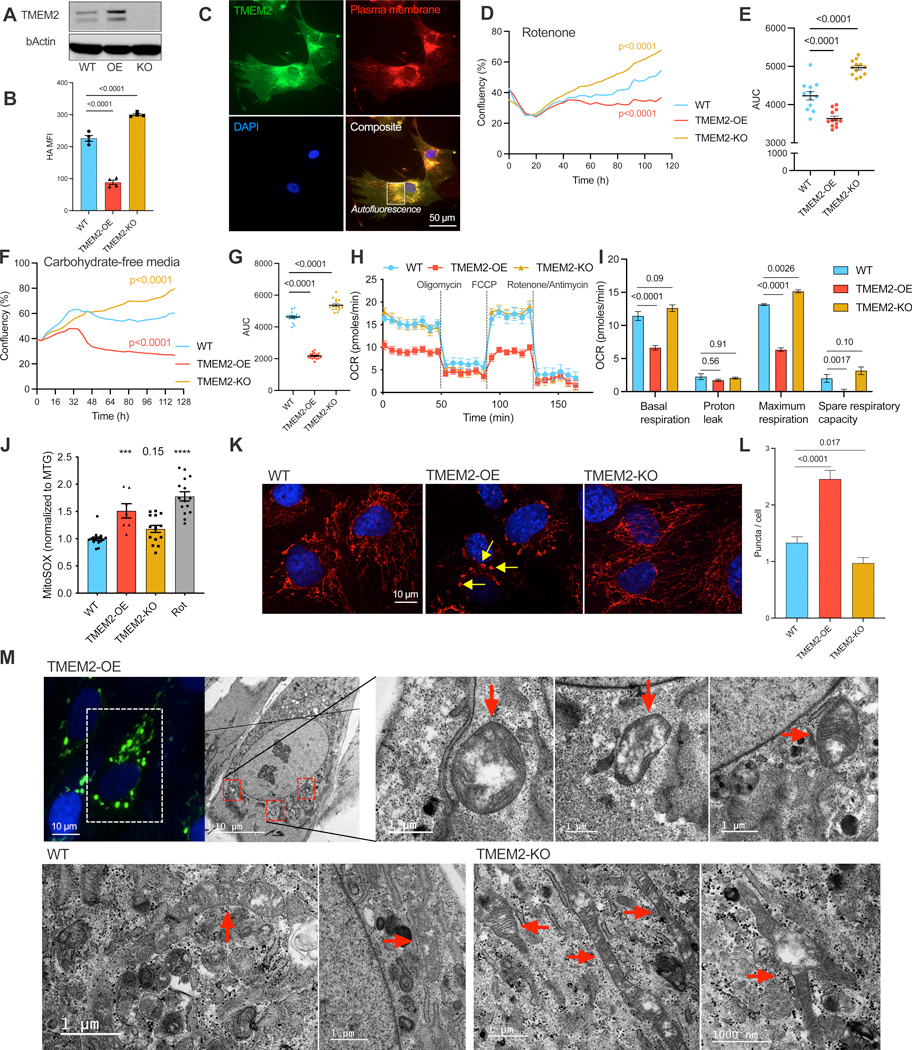
ECM remodeling results in altered mitochondrial function.

HA is a major GAG component pivotal for preserving the integrity and functionality of the mammalian ECM. To modulate HA homeostasis, we conducted both overexpression (OE) and knockout (KO) of the hyaluronidase TMEM2 in human fibroblasts, a cell type that generates and secretes a large amount of ECM components, including HA (Figure 1A, S1A/B), and is a sentinel that controls local immune responses during infection.^16^ TMEM2 localizes on the plasma membrane of fibroblasts and reduces the amount of HA in the ECM (Figure 1B/C), which is in line with its reported function as a cell-surface hyaluronidase that cleaves extracellular HA.^4^ Previous studies found that TMEM2 impacts cellular resistance against the endoplasmic reticulum (ER) stress inducer tunicamycin without affecting ER stress pathways,^17^ indicating that TMEM2 may regulate cellular proteostasis through an unknown mechanism. To assess how TMEM2-induced ECM remodeling may impact cellular homeostasis, we performed a series of stress resistance assays by treating cells with different stress inducers and measuring their growth using the Incucyte Live-cell Analysis System (Figure S1C/D). This dynamic growth-based assay is a highly quantitative physiological readout for measuring cellular resilience against different types of stress across a long period of time, and hence may reflect the quality of different cellular compartments. As a proof of concept, KO of the nuclear encoded mitochondrial electron transport chain genes, COX11 or SDHD, resulted in cells more sensitive to mitochondrial stress through exposure to rotenone (Figure S1E-G) or carbohydrate-free (CF) media (Figure S1H/I). Interestingly, alteration of the ECM, by overexpression of TMEM2, resulted in cells more sensitive to rotenone (Figure 1D/E) and CF media (Figure 1F/G, S1J, Video 1-3). Additionally, TMEM2-OE cells were also sensitive to a variety of other mitochondrial-stress conditions, including low glucose media (Figure S1K), galactose media (Figure S1L), NaN_3_ treatment that inhibits complex IV activities (Figure S1M), as well as the oxidative stress inducer H_2_O_2_ (Figure S1N). As a complementary approach to test whether HA-remodeling in the ECM can lead to mitochondrial stress, we treated wildtype cells with purified hyaluronidase and found that exogenous hyaluronidase treatment was sufficient to sensitize cells to rotenone (Figure S1O). In contrast, TMEM2-KO cells were more resistant to these mitochondrial stress conditions (Figure 1D-G, Figure S1J). Finally, we found little difference with stressors of the Golgi or the actin cytoskeleton (Figure S1P/Q), or inhibitors of general transcription or translation (Figure S1R/S).

Intrigued by the finding that alterations of the ECM sensitized cells to mitochondrial stress, we hypothesized that mitochondrial homeostasis might also be affected. Indeed, TMEM2-OE cells had a significant reduction of mitochondrial respiration rates, including both basal and maximum respiration (Figure 1H/I), which was associated with a slightly increased rate of glycolysis (Figure S1T). Moreover, TMEM2-OE cells had increased mitochondrial reactive oxygen species (ROS) (Figure 1J), and mildly reduced cellular ATP levels (Figure S1U). However, mitochondrial mass was not markedly altered in TMEM2-OE cells as assessed by both mitochondrial-protein ATP5A western blot analysis and MitoTracker Green flow cytometry staining (Figure S1V-X). TMEM2-KO cells did not have obvious functional enhancements compared to wildtype cells (Figure 1H/I), which may be due to already optimal mitochondrial functions in wildtype cells.

To gain a more direct understanding of mitochondrial changes occurring in TMEM2-OE and TMEM2-KO cells, mitochondrial morphology was analyzed using Airyscan confocal super-resolution microscopy. Surprisingly, mitochondria in TMEM2-OE cells form many enlarged puncta structures, whereas mitochondria in TMEM2-KO cells are more tubular and form very few puncta (Figure 1K/L). We first tested whether the mitochondrial puncta found in TMEM2-OE cells were mitophagy-digested mitochondrial aggregates. To do so, we tested if lysosomes, by following the lysosomal protein LAMP1, colocalized with the mitochondrial puncta. Very little co-localization was found (Figure S1Y). Next, correlative light and electron microscopy (CLEM) was performed to directly image these mitochondrial structures at nanometer resolution. CLEM analysis revealed that the puncta in TMEM2-OE cells are swollen individual mitochondria (Figure 1M), consistent with mitochondrial hyper-fission phenotypes found by electron microscopy.^18,19^ By contrast, TMEM2-KO cells had more elongated and branched mitochondria (Figure 1M), indicating hyper-fused mitochondria. Taken together, TMEM2-OE-induced ECM remodeling in human cells leads to increased mitochondrial fragmentation, declined mitochondrial respiration, increased mitochondrial oxidative stress, and increased sensitivity to mitochondrial stress.

### ECM remodeling results in altered mitochondrial function in *C. elegans*

**Figure 2.**
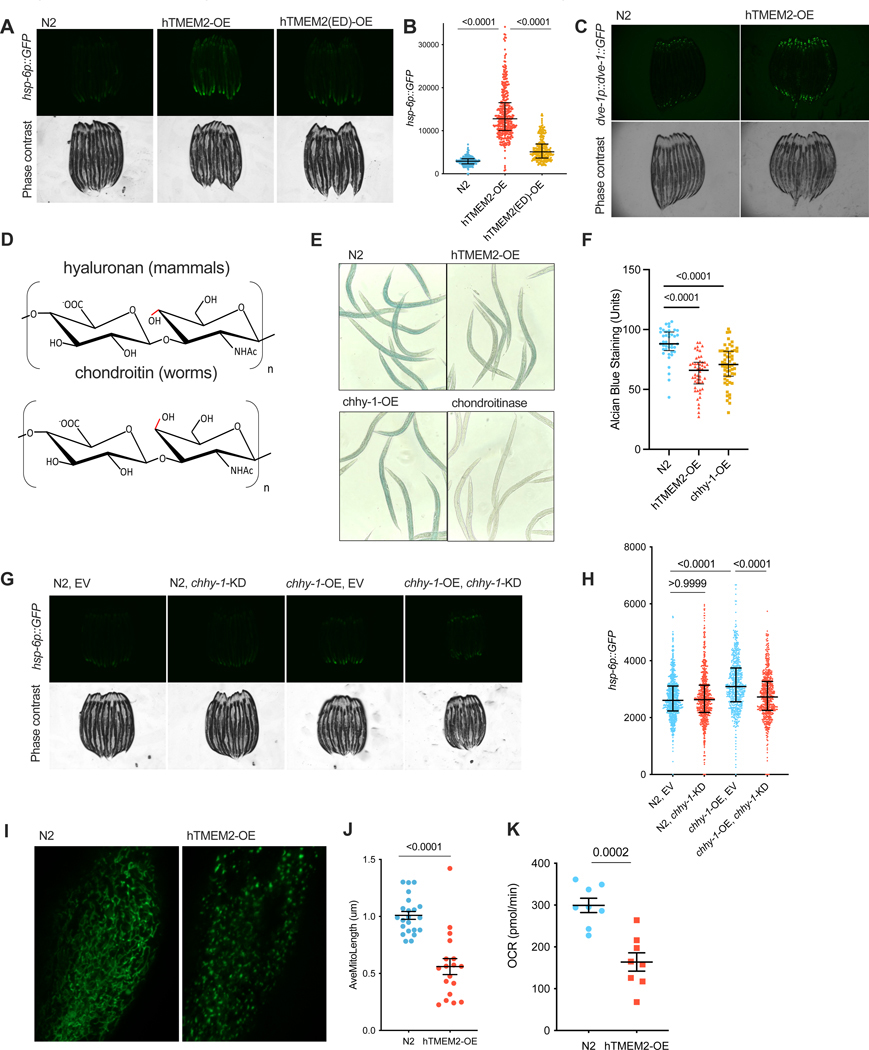
ECM remodeling results in altered mitochondrial function in C. elegans

Functioning as a pivotal organelle for environmental sensing and intercellular communication, the major ECM components are conserved from invertebrates to human.^20^ Therefore, it is possible that this phenomenon of ECM-mitochondria communication is conserved across species. To test this hypothesis, we ectopically over-expressed human TMEM2 (hTMEM2) in *C. elegans* and compared their transcriptome with wildtype worms. Multiple mitochondrial stress-related pathways including the UPR^MT^ were activated by hTMEM2 (Figure S2A). As an additional measure of mitochondrial stress responses induced by hTMEM2 overexpression, we tested if hTMEM2-OE could ectopically induce a known reporter gene of the UPR^MT^, *hsp-6*, using animals expressing the *hsp-6p::GFP* UPR^MT^ reporter. Overexpression of hTMEM2 significantly increased the expression of the *hsp-6p::GFP* reporter over 4-fold as assessed by fluorescence microscopy and quantified using a large particle flow cytometer (Figure 2A/B). Importantly, overexpression of a mutated version of TMEM2 lacking the HA depolymerization activity had no impact on the UPR^MT^ activity (Figure 2A/B)^4,17^, suggesting that hTMEM2 induces the UPR^MT^ by degrading components of the *C. elegans* ECM. Furthermore, hTMEM2 OE induces *hsp-6* expression in an *atfs-1*-dependent manner (Figure S2B/C) and results in robust nuclear localization of DVE-1 (Figure 2C), both hallmarks of *bona fide* UPR^MT^ induction.^21^

The extracellular matrix of *C. elegans* is made of chondroitin, an isomer of HA nearly identical except for the stereochemistry of the C-4 hydroxyl group on the hexosamine moieties (Figure 2D). Notably, many known hyaluronidases and chondroitinases exhibit overlapping substrate specificities.^22^ Overexpression of TMEM2 resulted in less chondroitin (Figure 2E/F). Likewise, *C. elegans* gene *chhy-1* encodes a chondroitinase.^23^ Similar to overexpression of TMEM2, overexpression of *chhy-1* reduced chondroitin levels (Figure 2E/F) and also induced the UPR^MT^ (Figure 2G/H). Therefore, hTMEM2 likely operates as a chondroitinase in *C. elegans* with CHHY-1 being its functional homolog.

To evaluate ECM-induced mitochondrial changes in *C. elegans*, we assessed the impact of hTMEM2-OE on mitochondrial morphology. Interestingly, hTMEM2-OE had no impact on mitochondrial morphology in young adult animals (Figure S2D/E). However, aged hTMEM2-OE animals exhibited pronounced mitochondrial fragmentation in comparison to wildtype N2 animals (Figure 2I/J). Consistent with observations in human cells, the oxygen consumption rate (OCR) also declined in hTMEM2-OE animals (Figure 2K).

Mitochondria are a major origin of cellular ROS, thereby frequently triggering oxidative stress and the accompanying oxidative stress responses amidst mitochondrial perturbations. In *C. elegans*, SKN-1, the ortholog of mammalian NRF2, is the key transcription factor that regulates oxidative stress responses. Using the SKN-1 target gene, *gst-4*, as a reporter, we found that hTMEM2 elicited an oxidative stress responses in a *skn-1*-dependent manner (Figure S2F/G), in alignment with our RNA-seq findings (Figure S2A).

Taken together, hTMEM2 induces similar mitochondrial functional declines as well as mitochondrial stress responses in *C. elegans* and human cells, indicating that this phenomenon of ECM-mitochondria communication may have early in evolution.

### The TGF-β receptor mediates communication between the ECM and mitochondria

**Figure 3.**
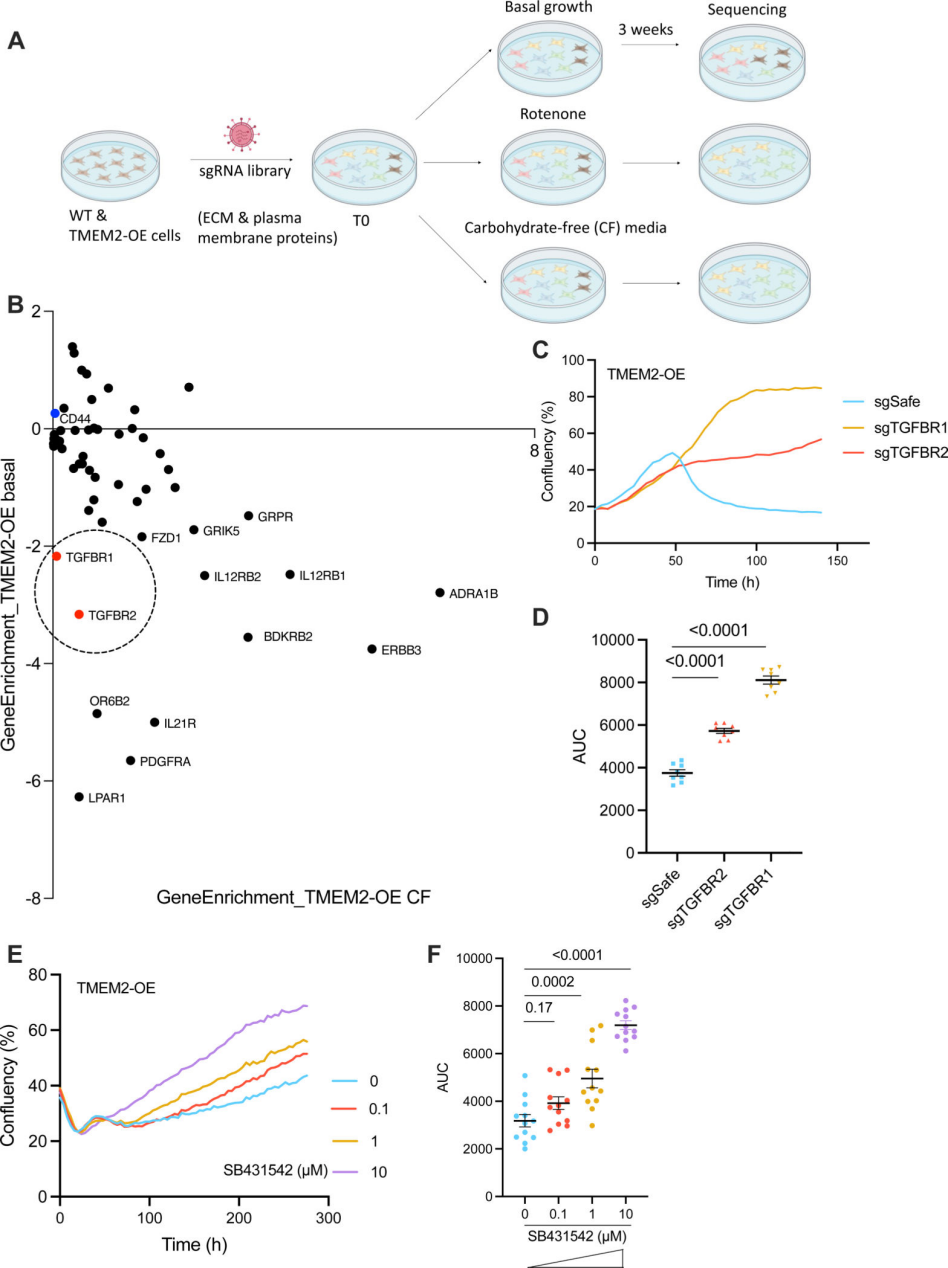
The TGF-β receptor mediates communication between the ECM and mitochondria

To uncover how changes in the ECM are communicated inside of the cell to alter mitochondrial form and function, we performed a CRISPR-KO screen targeting 2073 genes encoding proteins located on the plasma membrane (Supplementary Table 1). This library included ECM fibrous components such as collagens and elastin, proteoglycans that bind to or regulate ECM fiber, and cell-surface receptors pivotal for transducing signals from the extracellular milieu, including HA binding proteins. Following library transduction into Cas9-expressing wildtype or TMEM2-OE cells, we performed growth selection using rotenone or CF media in which TMEM2-OE cells are sensitive to (Figure 3A). Therefore, any gene identified in this CRISPR-KO screen would be expected to play a role in the communication from the ECM to mitochondria. In particular, genes whose loss increases the survival of TMEM2-OE cells in these mitochondrial challenged conditions would be predicted to mediate the TMEM2-induced mitochondrial remodeling.

As anticipated, the outcomes from rotenone- and CF media-mediated selections were highly congruent (Figure S3A), substantiating the robustness of this approach. To identify the signaling pathway downstream of TMEM2, we primarily focused on the signaling receptor subgroup of genes found in this screen. Particularly intriguing were the hits involving both subunits of the TGF-β receptor, TGFBR1 and TGFBR2 (Figure 3B). We validated that knocking out TGFBR1 or TGFBR2 was sufficient to rescue the growth deficiency phenotype of TMEM2-OE cells under conditions of mitochondrial stress (Figure 3C/D), as well as the use of the chemical inhibitor of TGFBR1, SB431542 (Figure 3E/F). Moreover, this screen generated a list of ECM genes that may regulate mitochondrial signaling in wildtype cells (Figure S4B-D). These provide a more comprehensive view on how ECM may impact mitochondria through various pathways.

To our surprise, we did not uncover integrin or HA-signaling pathways in the TMEM2-OE CRISPR-KO screen. The integrin signaling pathway converts mechanical cues from the ECM into regulatory signals and is known to stimulate mitochondrial stress signaling through mediators like FAK.^24,25^ The signaling role of HA is often mediated by various HA-binding proteins (HABPs) such as CD44 and LYVE1.^10^ We formally tested whether these pathways may regulate the TMEM2-mitochondria signaling. As a marker for integrin-signaling, FAK phosphorylation was measured, and no significant alterations were observed in TMEM2-OE and TMEM2-KO cells (Figure S4A-E), indicating that integrin activities might not be directly affected by TMEM2-induced ECM remodeling. This conclusion is further substantiated by findings in *C. elegans*, where hTMEM2 retained its capacity to robustly induce UPR^MT^ even when *pat-3,* the ortholog of human integrin-β,^25^ was knocked down (Figure S4F/G), indicating that integrins are not required for hTMEM2-induced mitochondrial remodeling in *C. elegans* either.

To test whether any HABPs may regulate TMEM2-induced mitochondrial remodeling, a targeted CRISPR-KO screen was performed, involving the knockout of each individual known HABP in wildtype cells, TMEM2-KO cells, and TMEM2-OE cells, followed by mitochondrial stress resistance assays (Figure S5A-D). None of the examined HABPs were essential for the hyper-sensitivity observed in TMEM2-OE cells or for the hyper-resistance in TMEM2-KO cells, except for one gene, P32 (Figure S5D). Indeed, P32-KO cells were sensitive to multiple mitochondrial stress (Figure S5E-J), indicating that P32 may preserve mitochondrial homeostasis. However, P32-KO did not suppress the rotenone-resistance phenotype induced by TMEM2-KO (Figure S5I/J), raising the possibility that P32 and TMEM2 may operate in parallel pathways. Moreover, P32 is mainly localized within mitochondria (Figure S5K) and is therefore unlikely to directly perceive and interact with HA in the ECM. These insights align with previous studies suggesting that P32 functions as a mitochondrial protein controlling mitochondrial translation.^26^

Taken together, TMEM2-induced HA remodeling in the ECM does not signal through integrins or HABPs to regulate mitochondrial homeostasis, but rather activate ECM-associated signaling pathways such as TGF-β.

### TMEM2 regulates mitochondrial homeostasis via the TGF-β-SMAD signaling pathway

The TGF-β-SMAD signaling pathways are highly conserved at the molecular and functional level from nematodes to humans with a well-documented role in regulating organismal development and immune responses.^27^ TGF-β ligands, constitutively secreted and sequestered in the ECM, undergo activation in response to mechanical or chemical disruptions of the ECM.^28^ Therefore, we hypothesized that TMEM2-induced ECM remodeling could be sensed by TGF-β, which subsequently signal to mitochondria in both nematodes and human cells via an evolutionarily conserved signaling pathway.

**Figure 4.**
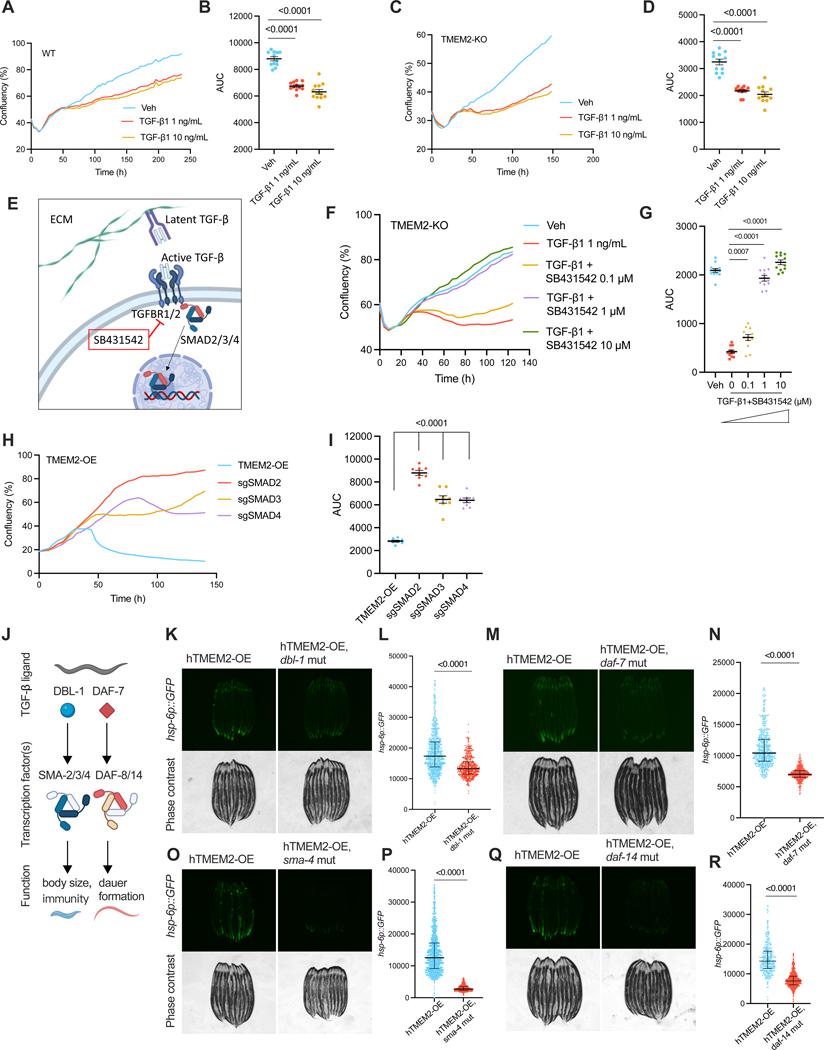
TMEM2 regulates mitochondrial homeostasis via the TGF-β-SMAD signaling pathway.

The TGF-β ligand superfamily is made up of over 30 members. To identify which TGF-β ligand regulates mitochondrial homeostasis in response to ECM alterations, we analyzed the transcriptome of BJ fibroblasts. TGFB1 emerged as the most abundantly expressed TGF-β ligand in BJ fibroblast cells, while TGFB2, INHBA, BMP1, BMP4, GDF11, and GDF15 exhibited lower expression levels (Figure S6A). If one of these TGF-β ligands was responsible for ECM-mitochondrial communication, exogenous addition of the TGF-β ligand to WT or TMEM2-KO cells would be predicted to sensitize these cells to mitochondrial stress, thus phenocopying TMEM2-OE cells. Consistent with this hypothesis, addition of TGF-β1 sensitized both wildtype and TMEM2-KO cells to mitochondrial stress (Figure 4A-D). Likewise, TGF-β2 mirrored this pattern by sensitizing cells to mitochondrial stress (Figure S6B/C). In contrast, BMP4, GDF11, and GDF15 showed little impact on cellular sensitivity to mitochondrial stress (Figure S6D-I).

Both TGF-β1 and TGF-β2 signal via the canonical TGFBR1/TGFBR2 receptor complex (Figure 4E).^27^ Loss of TGFBR1 function, using the TGFBR1 inhibitor SB431542, effectively suppressed the sensitizing effects of TGF-β1 and TGF-β2 on cellular vulnerability to mitochondrial stress in a dose-dependent manner (Figure 4F/G, S6J/K), indicating that TGF-β1 and TGF-β2 signal via the TGFBR to control mitochondrial homeostasis. More importantly, consistent with genetic knockout of TGFBR1 and TGFBR2 (Figure 3C/D), pharmacological inhibition of TGFBR using SB431542, improved the resistance of TMEM2-OE cells to mitochondrial stress (Figure 4F/G). Downstream of the TGFBR lies the canonical SMAD signaling pathway. Knocking out each member of the SMAD2/3/4 transcription factor complex was sufficient to protect TMEM2-OE cells against mitochondrial stress (Figure 4H/I). Therefore, TMEM2-induced ECM remodeling signals via the canonical TGF-β-TGFBR-SMAD signaling pathway to regulate mitochondrial homeostasis.

Intrigued by the finding that TGF-β signaling was necessary and sufficient for regulating mitochondrial homeostasis and functions downstream of TMEM2 alterations of the ECM, we asked if TGF-β signaling might be required for the communication of other forms of ECM remodeling for mitochondrial homeostasis. FBLN5 promotes the assembly of continuous elastin polymers and thus regulates the protein fiber organization of the ECM^29^. Consistent with a loss of ECM homeostasis, cells lacking FBLN5 were sensitive to mitochondrial stress and phenocopied the mitochondrial defects found in TMEM2 OE cells (Figure S6L-O). Furthermore, blocking TGF-β signaling with SB431542 improved the resistance of FBLN5-KO cells against mitochondrial stress (Figure S6N/O), indicating that FBLN5-associated ECM remodeling may also signal via the TGF-β-TGFBR signaling pathway. Therefore, TGF-β may have the capability to perceive diverse forms of ECM perturbations and subsequently employ the canonical TGFBR-SMAD signaling pathway to regulate mitochondrial homeostasis.

The TGF-β-SMAD signaling pathway is highly conserved across species. A total of five TGF-β ligands (*dbl-1*, *daf-7*, *unc-129*, *tig-2*, *tig-3*) have been identified that control developmental and immune responses of *C. elegans* (Figure 4J).^30^ To test which of these TGF-β ligands might regulate the hTMEM2-mitochondria pathway in worms, existing mutants of these ligands were combined with the *hTMEM2-OE, hsp-6p::GFP* strain. Notably, mutations of either *dbl-1* and *daf-7*, but not *tig-2* or *unc-129*, abrogated the induction of the UPR^MT^ in hTMEM2-OE worms (Figure 4K-N, S6P/Q). Moreover, *sma-4* (the worm orthologue of *SMAD4*) and *daf-14* (the worm orthologue of *SMAD2*) were required for hTMEM2-induced UPR^MT^ (Figure 4O-R). *sma-4* and *daf-14* were also required for hTMEM2-induced oxidative stress responses (Figure S6R-U), possibly through modulating mitochondrial homeostasis as well. Therefore, consistent with findings in human cells, the TGF-β signaling pathway also regulates mitochondrial homeostasis in response to hTMEM2 OE in *C. elegans*.

### TMEM2-induced TGF-β signaling induces mitochondrial fission

**Figure 5.**
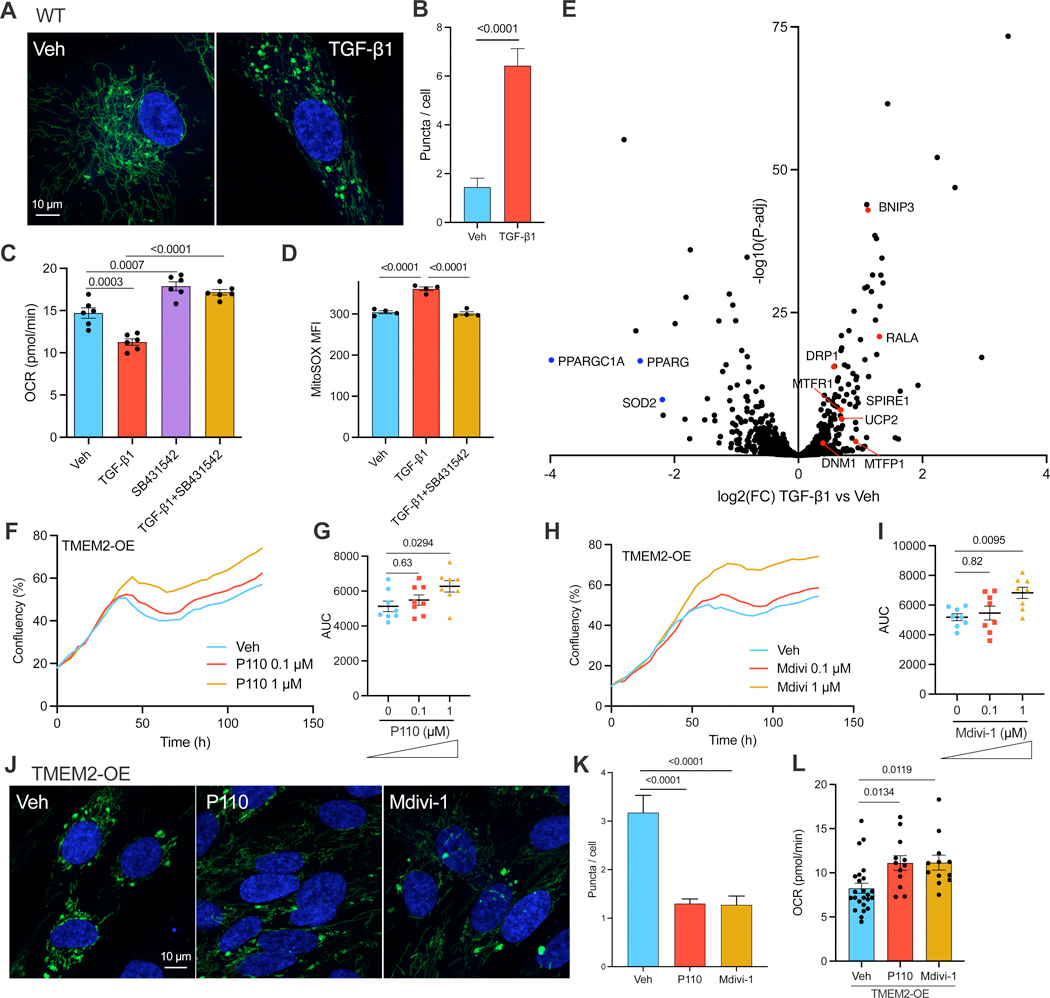
TMEM2-induced TGF-β signaling induces mitochondrial fission.

Based on our observations that TGF-β1 sensitizes cells to mitochondrial stress (Figure 4A-D), we hypothesized that TGF-β1 might directly alter mitochondrial form and/or functional changes in wildtype cells. Much like TMEM2-OE cells, TGF-β1 promoted the accumulation of enlarged mitochondrial puncta, indicative of augmented mitochondrial fragmentation (Figure 5A/B). When we assessed mitochondrial function, we found reduced OCR in TGF-β1-treated cells and this was rescued by the TGFBR inhibitor SB431542 (Figure 5C), indicating that the canonical TGF-β1 signaling pathway inhibits mitochondrial respiration. Notably, SB431542 alone also mildly increased the OCR of wildtype cells (Figure 5C), indicating that a low level of TGF-β1 signaling may be constitutively active in repressing mitochondrial functions under basal growth conditions. Moreover, TGF-β1 increased mitochondrial ROS levels, which was also attenuated by SB431542 (Figure 5D). Collectively, these findings indicate that TGF-β1 induces mitochondrial fission and functional deterioration similar to the effects observed with TMEM2-OE.

As demonstrated earlier, the SMAD pathway regulates mitochondrial stress responses in both human cells and nematodes (Figure 4). Therefore, we postulated that TGF-β1 might induce mitochondrial remodeling via the canonical SMAD pathway, potentially by inducing transcriptional alterations in specific mitochondrial genes. To test this hypothesis, RNA-sequencing (RNA-seq) analysis was performed on TGF-β1-treated cells. Remarkably, among the multitude of mitochondrial genes examined, several key genes associated with mitochondrial fission were up-regulated in response to TGF-β1, including DNM1, DRP1, UCP2, MTFP1, MTFR1, SPIRE1, RALA, and BNIP3 (Figure 5E). Indeed, the mitochondrial fission genes *DNM1*, *MTFR1*, and *RALA* are direct targets of SMAD3 in human embryonic stem cells,^31^ indicating that promoting mitochondrial fission may constitute one facet of the TGF-β1-SMAD program. Interestingly, anti-oxidative stress genes, including SOD2, PPARG, and PPARGC1A, were down-regulated by TGF-β1 (Figure 5E), in line with the increased generation of ROS observed in these cells.

To test whether mitochondrial fission plays a causative role in TMEM2-induced mitochondrial functional decline, we treated TMEM2-OE cells with two mitochondrial fission inhibitors, P110 and Mdivi-1. Either inhibitor significantly enhanced the resistance of TMEM2-OE cells to mitochondrial stress (Figure 5F-I). The mitochondrial morphology was restored by P110 or Mdivi-1 treatment, as indicated by reduced mitochondrial puncta in TMEM2-OE cells (Figure 5J/K). Moreover, the functional assessment of mitochondrial respiration in TMEM2-OE cells revealed a significant improvement in response to P110 or Mdivi-1 treatment (Figure 5L). Therefore, the TGF-β-SMAD signaling pathway may directly promote the expression of mitochondrial fission genes to modulate mitochondrial homeostasis, including increasing mitochondrial fission, reducing mitochondrial respiration, and increasing mitochondrial oxidative stress, in TMEM2-OE cells.

### TMEM2 promotes immunity through mitochondrial stress signaling

The ECM is an enormous structure that provides support for cells and is the first line of defense against pathogens in the earliest phases of infections. Furthermore, mitochondrial homeostasis is intimately linked with pathogenesis and many pathogens usurp essential mitochondrial metabolites for their own use.^32^ Therefore, an intriguing hypothesis has emerged from our findings that could link changes within the ECM to coordinate mitochondrial form and function to allow cells and organism to defend against pathogens.

**Figure 6.**
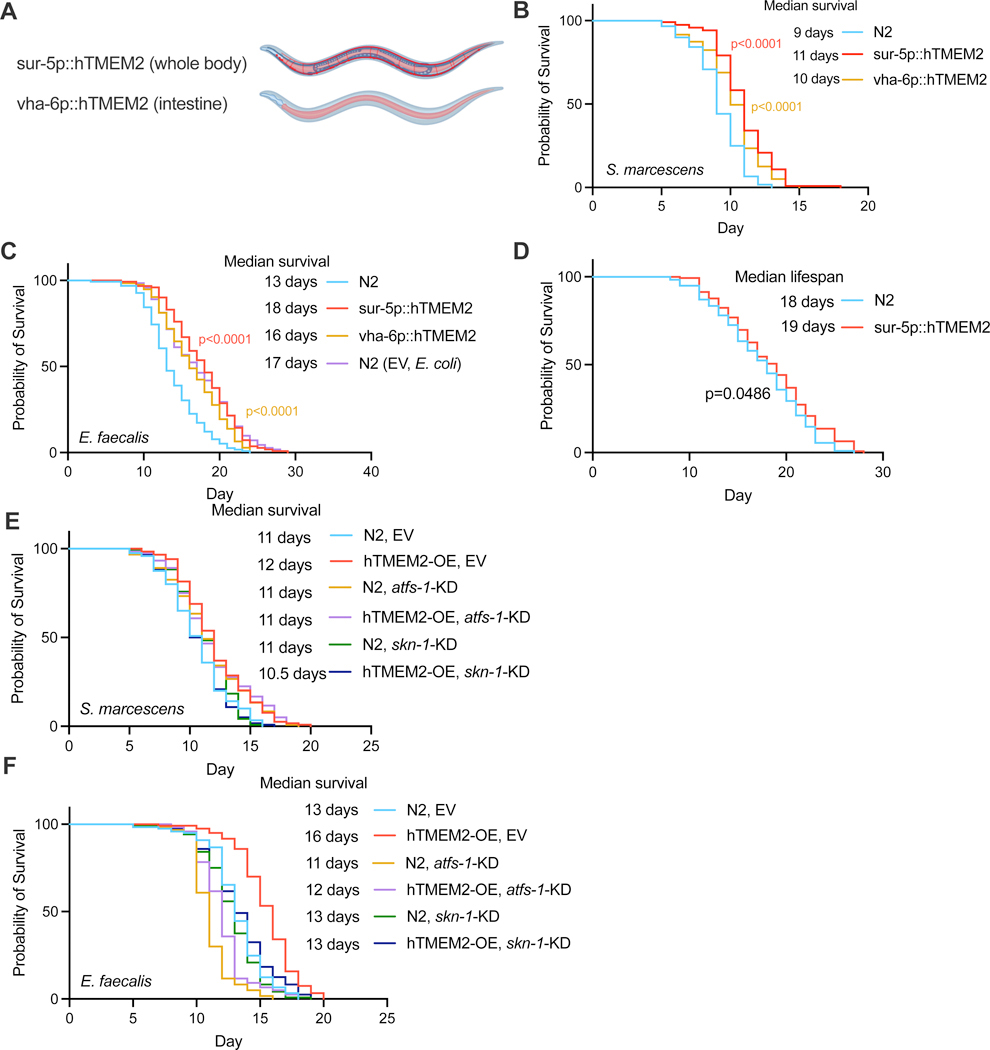
TMEM2 promotes immunity through mitochondrial stress signaling.

In their natural habitat, wild *C. elegans* consistently confront the risk of bacterial infection, given their reliance on bacteria as a food source. To investigate whether ECM remodeling-induced mitochondrial stress responses may enhance the immunity of nematodes, we performed pathogen-resistance assays using two human opportunistic pathogenic bacteria, *S. marcescens* and *E. faecalis*. Interestingly, overexpression of hTMEM2 (*sur-5p::hTMEM2*) exhibited a robust resistance to both types of pathogenic bacteria (Figure 6A-C), but had little effect on the lifespan on non-pathogenic *E. coli* (Figure 6D). Furthermore, restricted expression of TMEM2 to the intestine, the major organ in contact with the pathogenic bacteria, due to the dense cuticle surrounding the animal, significantly enhanced animal immunity against these pathogens (Figure 6A-C). Moreover, both ATFS-1-mediated UPR^MT^ and SKN-1-mediated oxidative stress response were indispensable for the enhanced immunity observed in hTMEM2-OE worms (Figure 6E/F). Taken together, these findings underscore that hTMEM2-induced ECM remodeling and the associated mitochondrial stress responses may offer protection against infections.

## Discussion

In our previous work, we found that TMEM2 promotes cellular resistance against the ER stress inducer tunicamycin.^17^ However, this protective effect was not mediated through the ER unfolded protein responses (UPR^ER^).^17^ Therefore, we speculated that TMEM2 may signal to regulate other aspects of cellular stress responses rather than directly controlling ER functions. This was tested by various types of stress assays, leading to the discovery that mitochondria are directly regulated by TMEM2 signaling. Specifically, TMEM2-induced ECM remodeling leads to mitochondrial fragmentation and functional declines in both human fibroblasts and *C. elegans*. We eliminated the possibility of HA directly transmitting signals to mitochondria via HABPs. Instead, through a set of genetic screens, we identified the TGF-β signaling pathway as one mediator of TMEM2-induced mitochondrial remodeling by promoting mitochondrial fission. Physiologically, this ECM-mitochondria communicating mechanism may confer specific benefits to animal immunity in a tissue-specific manner.

The most important aspect of this body of work is that it reveals that the ECM has an “alarmin” aspect to it that involves the release of a stored ligand, TGF-β, that acts through its receptor and the downstream SMAD signaling complex to alter mitochondrial form and function. It is intriguing to speculate that during the evolution of eukaryotes, systems originally aimed to protect bacteria before endosymbiosis, such as morphological changes, were handed off to the host cell to be actively engaged during conditions of pathogen infections, such as bacterial infections, to now alter mitochondrial morphology and stress responses. As the pathogenic bacteria made its way through the ECM, alarmins, such as TGF-β, are released to protect the cell from the invading pathogen via remodeling its mitochondria. Indeed, many bacterial pathogens target eukaryotic cells for their mitochondrial rich resources, such as iron, in the case of *Pseudomonas aeruginosa* infection and other siderophore-producing bacterial infections.^33,34^ The ability of mitochondria to divide themselves to create smaller quanta of useable resources by the invading bacteria might be one form of early protection from pathogens and this is mediated by cues from alterations in the ECM.

In our study we found that alterations of the ECM by either TMEM2 overexpression of loss of FBLN5 resulted in the same TGF-β-mediated signaling and downstream mitochondrial changes. It is interesting to speculate whether all changes of the ECM signal to the cell through TGF-β signaling, or whether there will be multiple ECM-derived alarmins. If so, will each alarmin have a distinct set of pathogens that it responds to, thus tailoring the defense modes required for the cell to tolerate and survive the pathogen? Lastly, our work has been focused on fibroblast cells, which are among sentinel cells first encountering pathogens, whereas our *C. elegans* studies became focused on the intestinal cells, one of the few cell types in direct contact with the environment. In the future it will interesting to learn if other cell types, especially innate immune cells have the same signaling mechanism and possibly more complex systems to signal their ECM changes in a cis- and/or trans-manner.

Finally, this work has identified one of the very first ligand-receptor interactions on the plasma membrane that regulates mitochondrial form and function. Changes to mitochondrial morphology have mainly been driven by intercellular signals, such as fission driven by either PINK1/PARKIN interactions on the mitochondrial surface or DRP1 driving mitochondrial division.^35^ Therefore, our findings provide a new direction to change mitochondrial function by changing the outside of the cell, the ECM, to drive mitochondrial changes.

The integrity of HA has long been associated with mammalian longevity. The naked mole rat (NMR, *Heterocephalus glaber*) is an extremely long-lived species with a maximal lifespan exceeding 30 years. In comparison, laboratory rats (*Rattus norvegicus*) with a similar body weight have a lifespan capped at 4 years. One prominent molecular hallmark of NMR cells is the presence of very high molecular weight HA in the ECM, ranging 6,000–12,000 kDa, comparing to 500–3,000 kDa in humans and mice.^36^ Remarkably, ectopic overexpression of the NMR HA synthase gene *Has2* (nmrHas2) in mice is sufficient to increase HA deposition in multiple tissues, and prolong the lifespans possibly via reducing inflammation with age.^37^ Along with our observations that TMEM2-KO fibroblasts are resistant to mitochondrial and oxidative stress, *nmrHas2*-overexpressing mouse cells also exhibit heightened resistance to H2O2-induced oxidative stress. Combining the two studies, a more comprehensive model can be formulated: Preserving HA or ECM integrity suppresses immune activation and inflammation to prolong mammalian lifespans, whereas activating ECM-mitochondria signaling can boost immunity across species. An intriguing avenue for future exploration involves manipulating HA integrity in different tissues to achieve an optimal outcome, *i.e.* extending mammalian lifespans by increasing HA integrity to reduce basal inflammation, while enhancing immune activation efficiency through controlled reduction of HA integrity during infections or vaccinations.

The skin and mucous membranes are the first defense mechanism of the immune system. Breaching this barrier is often the initial step during infection. This breach is facilitated by various ECM-degrading enzymes, including hyaluronidases, collagenases, and elastases, which are secreted by bacteria as key virulent factors.^38^ Cell-surface pattern recognition receptors, such as toll-like receptors (TLRs), are evolutionarily conserved frontline sensors for these microbes through recognizing bacteria-specific structural components, such as lipopolysaccharides and flagellin.^39^ On the other hand, immune responses are not exclusively triggered by pathogens. Other forms of tissue damage also elicit immune responses for repair. A common feature between external pathogen invasion and internal tissue damage is the degradation of the ECM. From this perspective, our findings introduce a novel mode of first-line innate immune activation, where ECM damage is directly sensed by ECM-bound factors, such as TGF-β, leading to a cascade of mitochondria-based signaling events that activate immune responses coordinated with metabolic remodeling. This pathway operates without the need for specific pattern recognition receptors and hence may have broader implications in tissue damage or other developmentally related ECM remodeling events that are associated with mitochondrial alterations.

## Supplementary Material

Supplementary Fig 1

Supplementary Fig 2

Supplementary Fig 3

Supplementary Fig 4

Supplementary Fig 5

Supplementary Fig 6

Supplementary Table 1

Supplementary Table 3

Supplementary Table 2

Supplementary Video 1

Supplementary Video 2

Supplementary Video 3
