# Supplementary figures and images for: The extracellular matrix integrates mitochondrial homeostasis
Source: Cell. Author manuscript; Available in PMC 2025 Aug 15. (PMC12352124; doi:10.1016/j.cell.2024.05.057)

Figure S1. TMEM2 sensitizes cells to mitochondrial stress. Related to Figure 1.

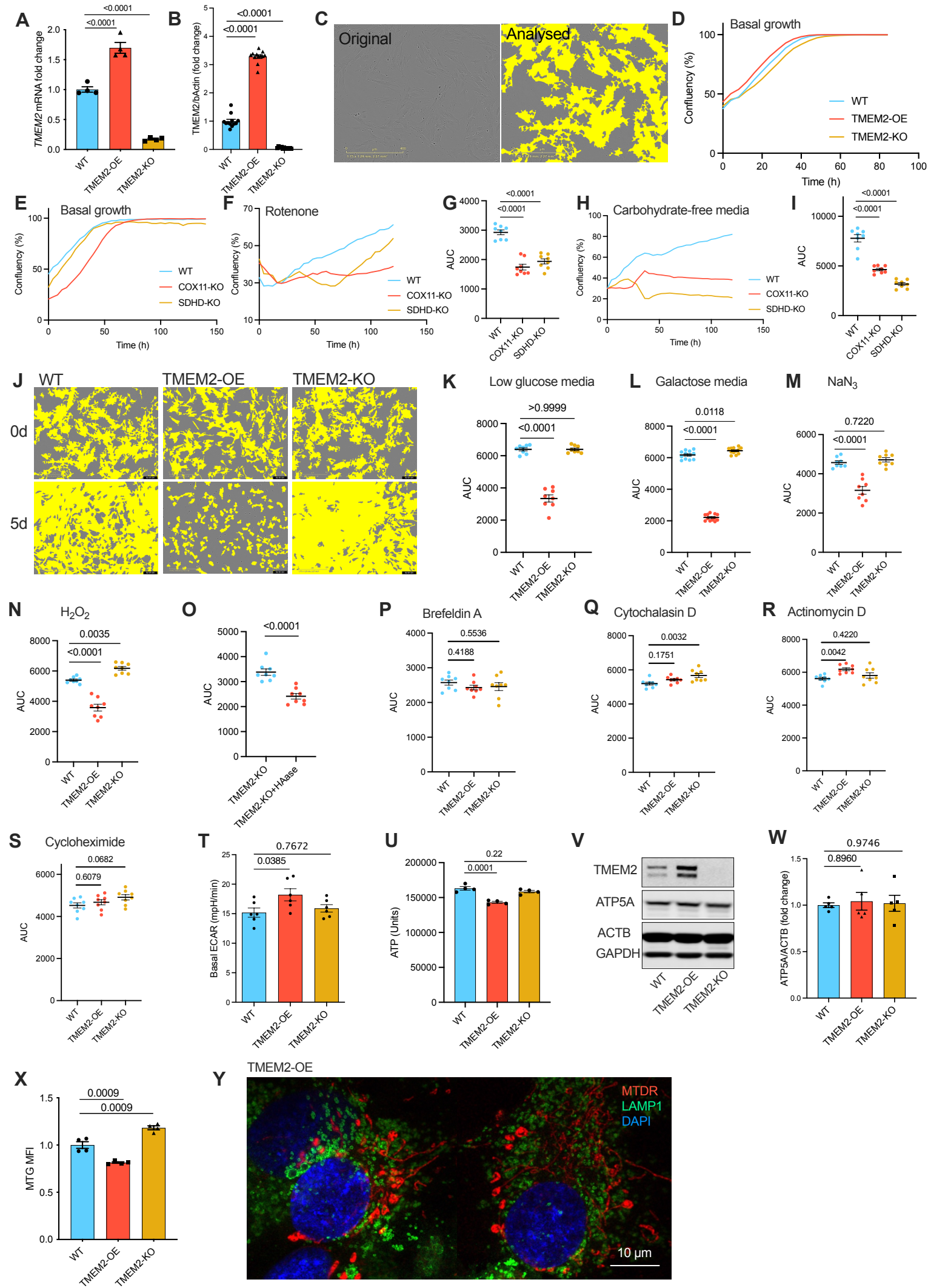

Supplement: Supplementary Fig 1 [file NIHMS2092591-supplement-Supplementary_Fig_1.pdf]

Figure S2. Human TMEM2 induces UPR<sup>MT</sup> in an *atfs-1*-dependent manner. Related to Figure 2.

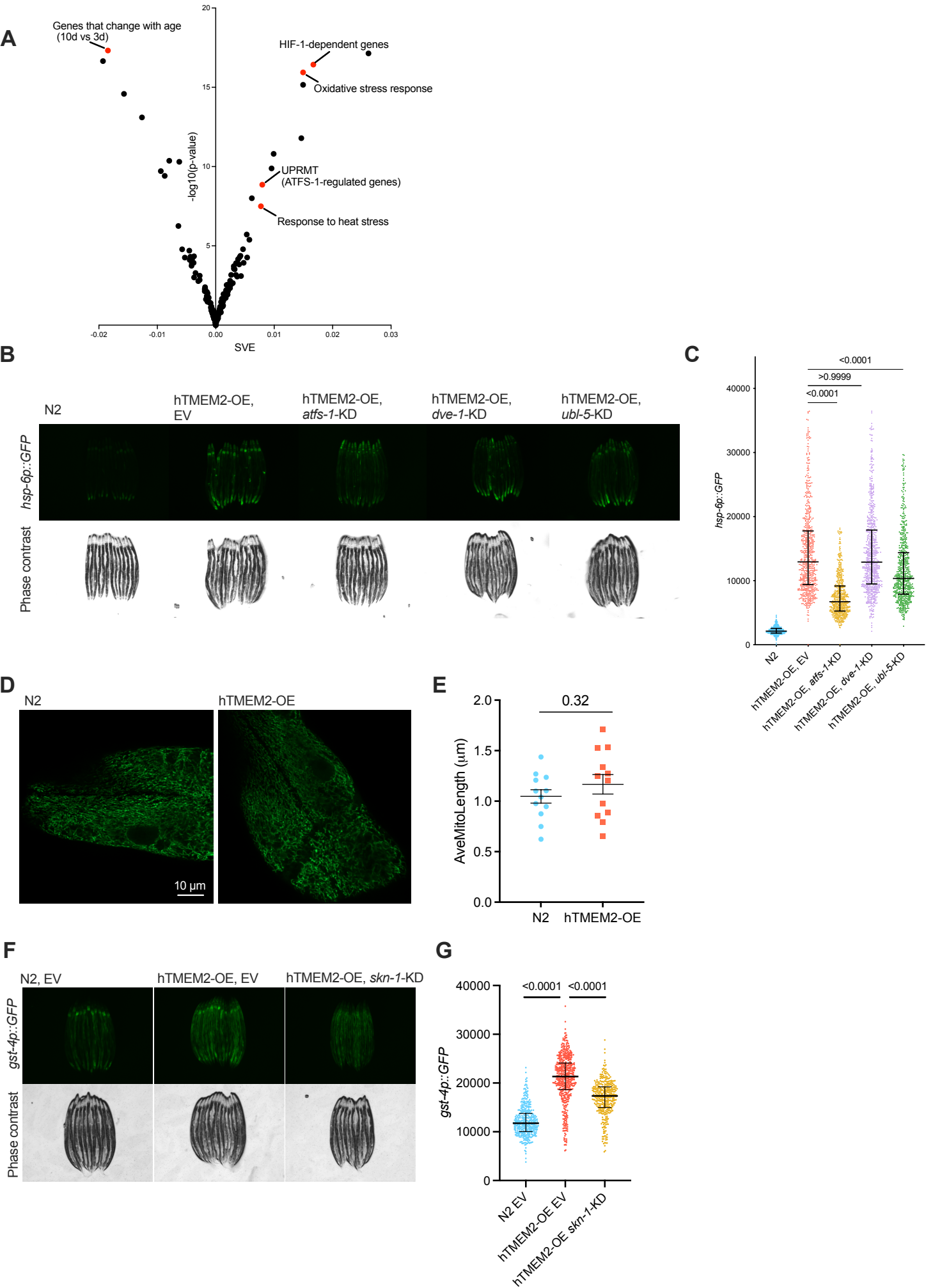

Supplement: Supplementary Fig 2 [file NIHMS2092591-supplement-Supplementary_Fig_2.pdf]

Figure S5. HABPs do not mediate the TMEM2-mitochondria crosstalk. Related to Figure 3.

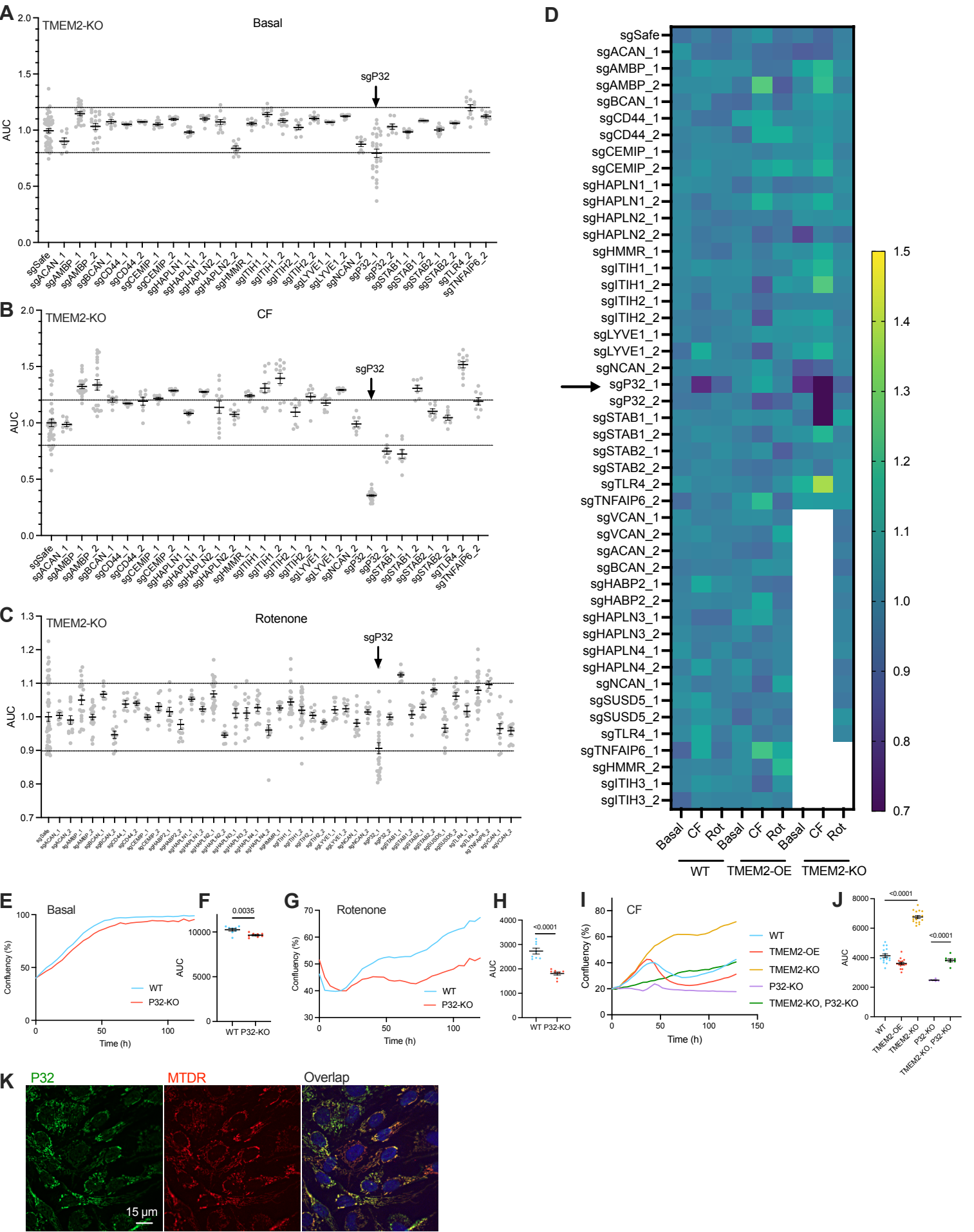

Supplement: Supplementary Fig 5 [file NIHMS2092591-supplement-Supplementary_Fig_5.pdf]

Figure S6. TGF- $\beta$  regulates mitochondrial homeostasis. Related to Figure 4.

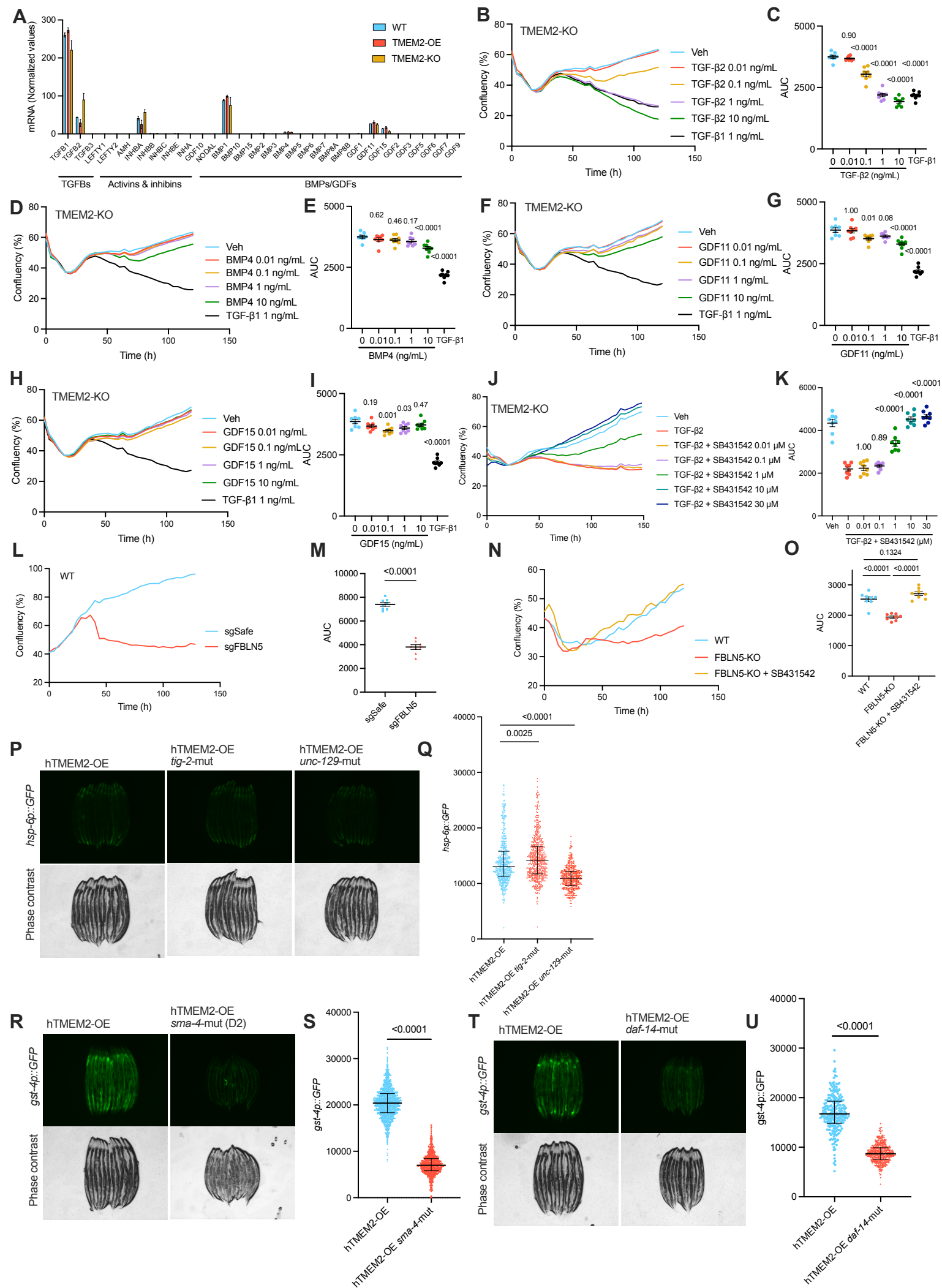

Supplement: Supplementary Fig 6 [file NIHMS2092591-supplement-Supplementary_Fig_6.pdf]
