# Supplementary material for: The extracellular matrix integrates mitochondrial homeostasis
Source: Cell. Author manuscript; Available in PMC 2025 Aug 15. (PMC12352124; doi:10.1016/j.cell.2024.05.057)

Figure S3. Novel mitochondria-regulating genes identified by a CRISPR-KO screen. Related to Figure 3.

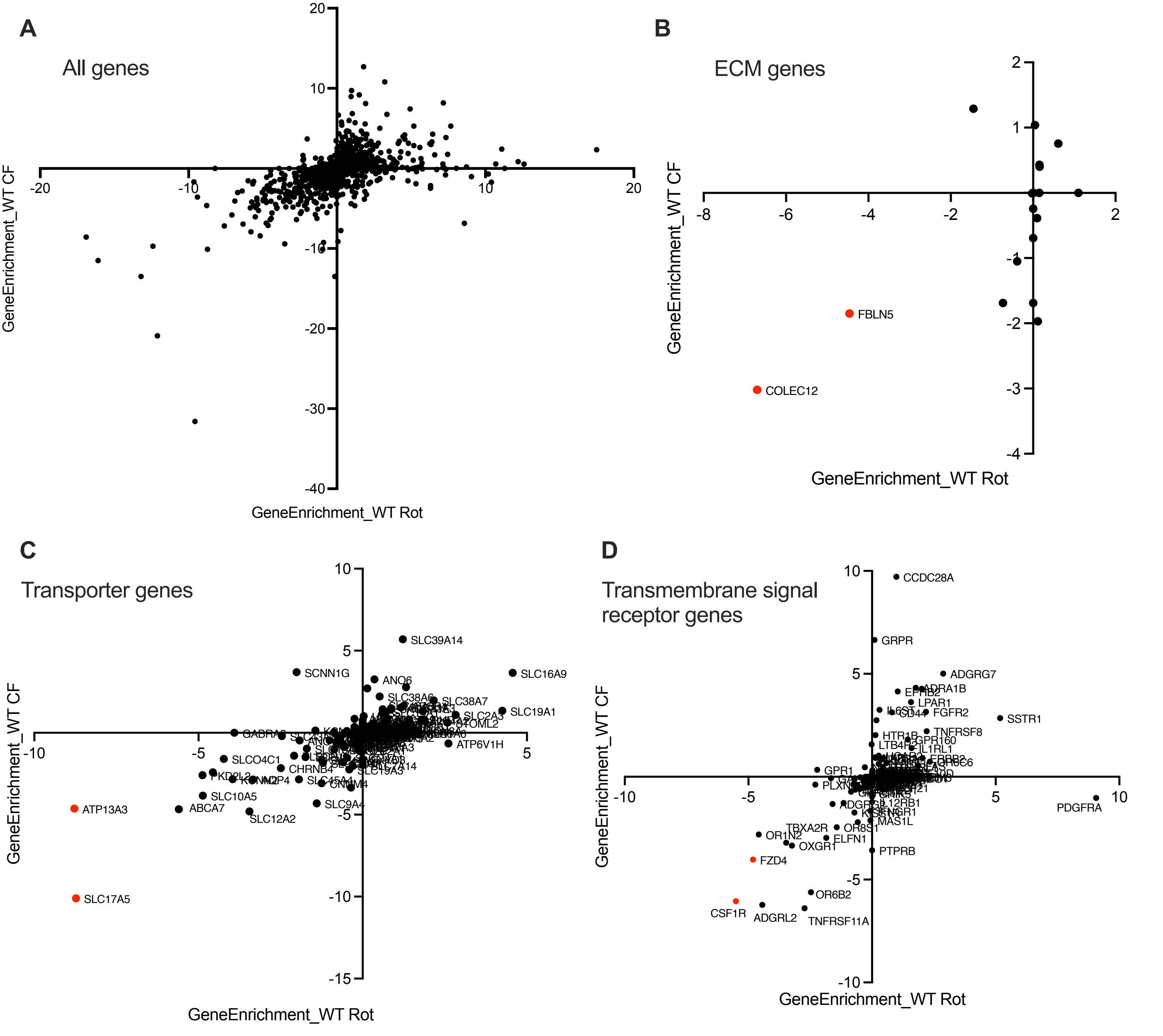

Supplement: Supplementary Fig 3 [file NIHMS2092591-supplement-Supplementary_Fig_3.pdf]
