# Supplementary material for: The extracellular matrix integrates mitochondrial homeostasis
Source: Cell. Author manuscript; Available in PMC 2025 Aug 15. (PMC12352124; doi:10.1016/j.cell.2024.05.057)

Figure S4. TMEM2 does not signal via the integrin signaling pathway to impact mitochondria. Related to Figure 3.

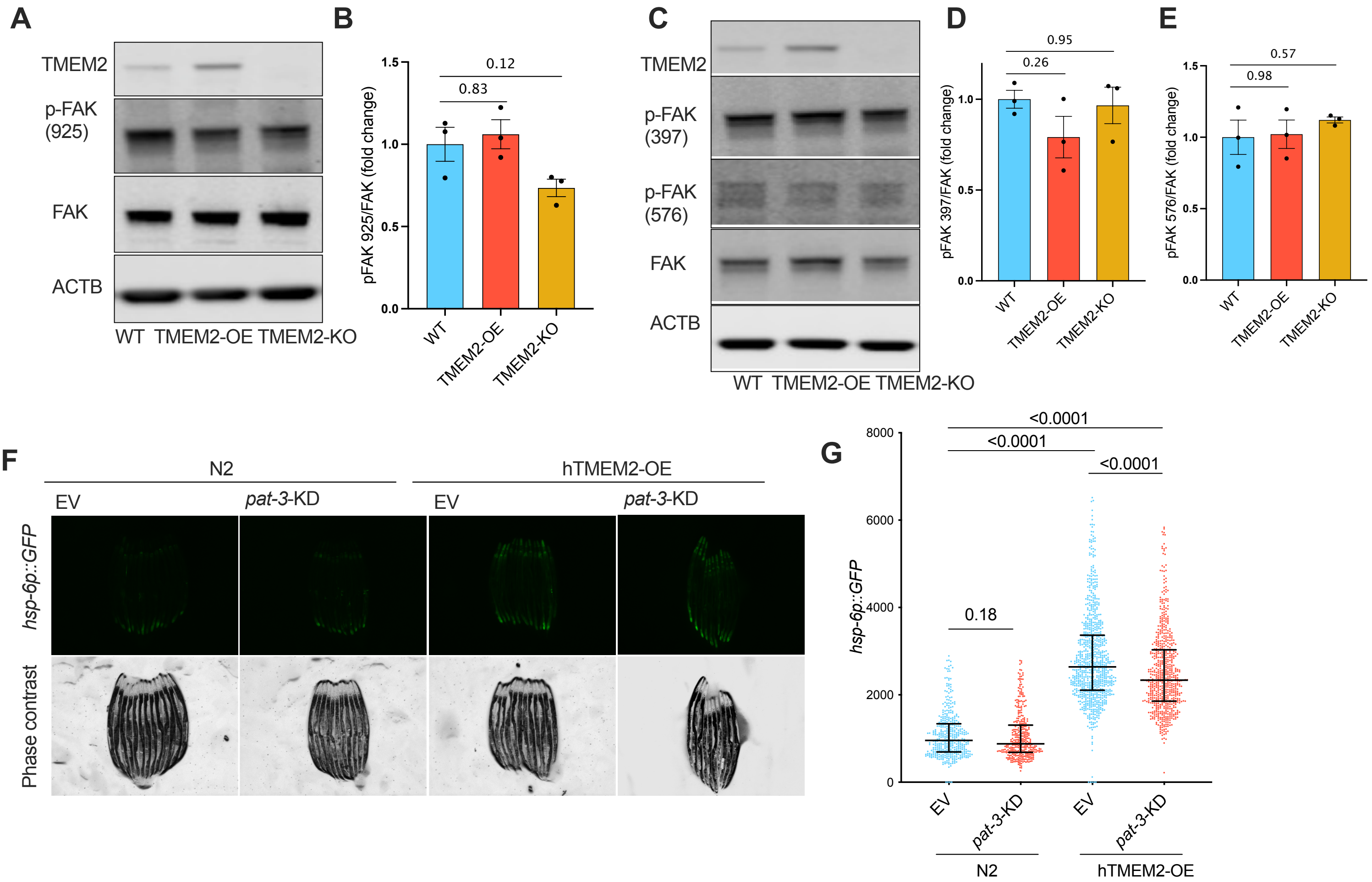

Supplement: Supplementary Fig 4 [file NIHMS2092591-supplement-Supplementary_Fig_4.pdf]
